# Implementation of stethoscope disinfection: an observational study on nursing staff practice and knowledge

**DOI:** 10.3205/dgkh000485

**Published:** 2024-05-17

**Authors:** Seda Şahan, Sevil Güler, Emine Korkmaz

**Affiliations:** 1 İzmir Bakircay University, Health Sciences Faculty, Nursing Department, İzmir, Turkey; 2 Erciyes University, Health Sciences Faculty, Nursing Department, Kayseri, Turkey; 3 Kayseri City Hospital, Kayseri, Turkey

**Keywords:** stethoscope, nurses, disinfection, practice, Turkey

## Abstract

**Background:**

Healthcare-associated infections cause high mortality and morbidity, and lack of stethoscope disinfection is one of the reasons for healthcare-associated infections. Nurses who frequently use stethoscopes in the clinic do not disinfect stethoscopes at high rates. This study aimed to identify the frequency of stethoscope disinfection by nurses and their knowledge about the same.

**Methods:**

This was a mixed-methods observational study. The quantitative part of the study included 202 nurses, the qualitative part included 12. Two researchers who made observations during stethoscope use recorded the procedures the nurses performed on the “Observation Form”. Semi-structured in-depth interviews were conducted based on phenomenological methods.

**Results:**

23.7% of the nurses disinfected their stethoscopes before contact with patients, 11.8% after contact with patients and 6.4% before and after contact with patients. The nurses used a stethoscope on an average of 7.42 patients without disinfecting it. In the qualitative interview, some nurses stated that they did not have information about the disinfectants to be used for stethoscopes and their effectiveness. Some of the participants in the present study stated that they did not receive training on stethoscope disinfection and that they did not know that there were guidelines about it.

**Conclusion:**

Since there were deficiencies in the implementation of stethoscope disinfection as well as knowledge, the transfer of knowledge in this context must receive more attention in education and training.

## Zusammenfassung

**Hintergrund:**

Healthcare-assoziierte Infektionen verursachen hohe Mortalität und Morbidität; einer der Gründe ist die unterlassene Desinfektion von Stethoskopen. Da Krankenschwestern und -pfleger, die in der Klinik häufig Stethoskope verwenden, häufig die Stethoskope nicht desinfizieren, sollten die Häufigkeit der Desinfektion von Stethoskopen und das Wissen darüber ermittelt werden.

**Methoden:**

Es handelte sich um eine Beobachtungsstudie. In den quantitativen Teil der Studie wurden 202 Krankenschwestern einbezogen, in den qualitativen Teil 12 Krankenschwestern. Zwei Untersucher, die die Stethoskopdesinfektion beobachteten, hielten die von den Krankenschwestern durchgeführten Verfahren in einem Beobachtungsformblatt fest. Im qualitativen Teil wurden halbstrukturierte Tiefeninterviews auf der Grundlage phänomenologischer Methoden durchgeführt.

**Ergebnisse:**

Es wurde festgestellt, dass 23,7% der Pflegenden ihre Stethoskope vor dem Kontakt mit dem Patienten, 11,8% nach dem Kontakt mit dem Patienten und 6,4% (n=13) vor und nach dem Kontakt mit dem Patienten desinfizierten. Durchschnittlich benutzten die Krankenschwestern ein Stethoskop bei 7,42 Patienten ohne zwischenzeitliche Desinfektion.

Aus den Interviewergebnissen geht hervor, dass einige Pflegende angaben, sie hätten keine Informationen über die bei der Stethoskopreinigung zu verwendenden Desinfektionsmittel und deren Wirksamkeit. Einige gaben an, dass sie keine Schulung zur Stethoskopdesinfektion erhalten hatten und nicht wussten, dass es diesbezüglich Richtlinien gibt.

**Schlussfolgerung:**

Da es sowohl Mängel in der Durchführung der Stethoskopdesinfektion als auch diesbezügliche Wissensdefizite gab, ist die Wissensvermittlung hierzu in der Aus- und Weiterbildung einschließlich der Schulung entsprechend zu berücksichtigen.

## Introduction

Healthcare-associated infections (HAIs) cause significant patient mortality and morbidity [1]. Multiple factors contribute to the occurrence of HAIs and patient-to-patient transmission. While hand hygiene is frequently emphasized in the context of HAIs, the significance of stethoscope use and disinfection, a potential source of infection transmission, often receives limited attention [2], [3]. The stethoscope stands out as one of the most commonly utilized medical devices in healthcare settings, and is integral to diagnostic procedures and care practices such as physical examinations and blood pressure measurements. Its non-invasive nature makes it a preferred choice due to accessibility, cost-effectiveness, and safety [4]. However, studies have shown that stethoscopes are contaminated by infectious pathogens such as methicillin-resistant *Staphylococcus aureus* (MRSA), vancomycin-resistant enterococci (VRE), and Klebsiella spp. [3], [5], [6]. Numerous studies conducted across the globe have reported a high contamination rate of stethoscopes, ranging from 66%–100% [7]. Observational studies report much lower stethoscope disinfection rates in the range of 11% to 16% [8], [9]. In one study of 358 healthcare providers (attending physicians, medical students, nurses, and residents), 76% used their stethoscopes frequently at work. Although 93% of them were aware of the possibility of stethoscope pathogen transmission, only 29% reported stethoscope disinfection after every use [10]. Cited barriers to hygiene performance include lack of time and poor access to disinfecting materials. These barriers persist despite most healthcare workers being aware that stethoscopes can be contaminated with pathogens and potentially serve as vector for transmission [11].

It has also been reported in the literature that stethoscopes and hand surfaces have similar contamination levels after physical examination of a patient, and pathogens on the stethoscope can be transmitted to patients during contact [9], [12], [13]. Despite this well-known fact, there are no clear guidelines on stethoscope disinfection. The Centers for Disease Control (CDC) categorize the stethoscope as a “non-critical medical device” and recommends disinfection after each patient contact. If the stethoscope is contaminated with blood, e.g., in hemodialysis, a tuberculocidal disinfectant or a disinfectant with specific label claims for HBV and HIV should be used (e.g., 1:100 dilution of a hypochlorite solution [500–600 ppm free chlorine]) [14].

In a study by Napolitani et al. [15], it was noted that bacterial contamination on the stethoscope surface could be effectively disinfected by alcohol-based disinfectants and alcohol wipes. It is also reported that hydrogen peroxide and alcohol-based wipes, which are widely available in hospitals, are effective and useful for stethoscope disinfection [16], [17].

Several studies have investigated the timing and frequency of stethoscope disinfection by nurses. A study found that 2.9% of the nurses disinfected the stethoscope before use on the patient and 1.5% thereafter [12]. A study in Nepal identified that 6.89% of the nurses disinfected the stethoscope after each use [18], while 24% of the nurses in the USA [11] and 37.7% of the nurses in Pakistan disinfected the stethoscope after contact with the patient [19].

This is a mixed-methods observational study aimed at uncovering the knowledge and practices of nurses regarding stethoscope disinfection in two hospitals in Turkey.

## Methods

### Ethics approval

Before starting the study, ethical approval was obtained from the ethics committee of the İzmir Bakircay University (no: 801, date: 21/12/2022). Institutional permission was obtained from the hospital where the research was carried out (date: 06/10/2022). All the procedures were performed in agreement with the ethical guidelines from the Declaration of Helsinki. Informed consent was obtained from the participating nurses.

### Study design

This mixed-methods study collected observational, quantitative and qualitative data. Because to the authors’ knowledge the literature does not contain any study on nurses’ disinfection of stethoscopes and the obstacles to doing so, obstacles were also emphasized, especially by adding qualitative interviews. In addition, the relationship between nurses’ knowledge and practices was examined using observational and quantitative methods: the nurses’ stethoscope disinfection practices were observed, and data were collected from 12 participants through a questionnaire to qualitatively assess their knowledge level on the topic, which was intended to support the analysis and understanding of the quantitative findings (based on 202 participants). The 12 questionnaire participants were selected based on predetermined criteria [20], [21].

### Participants

The study’s population comprised nurses employed in hospitals situated in two provinces (İzmir and Kayseri) within Turkey’s two different regions (Aegean and Central Anatolia Region) The sample size for the quantitative part of the study was determined using the G Power Analysis program based on the sample numbers of similar studies [3], [12], [22]. According to the calculation, 202 participants had to be included for 95% power. 

For the questionnaire (qualitative data), participants were selected using the purposive sampling method. Sampling was performed to maximize variation; thus, nurses with various occupational and demographic characteristics were selected to provide a wide range of information. As there are no strict rules (such as interviewing a certain percentage of the entire sample) in qualitative research, the qualitative part was completed with 12 nurses who volunteered, since it was thought that data saturation was reached regarding the research topic [23]. To ensure that the volunteered participants had the relevant experience and expertise, the criterion “use of a stethoscope”. In addition, care was taken to ensure the diversity of nurses in terms of demographic characteristics. Nurse who did not meet these criteria were excluded from the study. This careful selection process ensured that participants were of different ages, professional experience, and workloads at the clinics where they worked. In this way, data duplication was prevented by interviewing nurses with the same qualifications. It also ensured data diversity in qualitative interview results.

### Data collection

The data were collected through open-ended, in-depth, semi-structured, face-to-face interviews. Open-ended questions in the interview guide allowed the respondents to explain their own experiences, and follow-up questions were asked based on the participant’s responses. The data were collected from January to September 2023.

### Data collection tools

The individual information form consisted of 5 questions about the nurses’ age, gender, seniority in the profession, working years in the clinic, and educational status.

The knowledge and attitude form was prepared in line with the literature [9], [12], [24], [25] to determine nurses’ knowledge level and attitudes regarding stethoscope disinfection. The form consists of 10 questions related to the frequency of stethoscope disinfection, the time point of disinfection (before or after patient contact), the material used for disinfection and the relationship between health care-associated infections and stethoscope use. A different form, consisting of 4 questions on attitude toward and knowledge about stethoscope disinfection, was applied as asemi-structured interview. The authors sought expert opinions from 10 individuals who had a doctorate degree or at least 5 years of clinical experience on the questions to be included in the semi-structure interview. The final questions were:

1. What do you know about stethoscope disinfection?
2. What are your obstacles to disinfect of stethoscopes in the clinic?
3. Do you know the guidelines for disinfecting non-medical devices such as stethoscopes?
4. Do you think a stethoscope could be a source of infection?

### Data collection stages

Data were collected in 3 stages. Stage 1: observation; stage 2; application of the quantitative 10-item questionnaire: stage 3: qualitative data collection via the 4-item semi-structured interview.

The first stage of the study included observing the routine procedures in which nurses used a stethoscope, such as blood pressure measurement, and other non-routine procedures in which they used a stethoscope, depending on their working hours.

During stethoscope use, observations were made by a second researcher and a nurse who had completed her doctorate working as a specialist nurse in the clinic. The two researchers who made observations during stethoscope use recorded the procedures the nurses performed on the 10-item observation form. The researchers engaged in the observation stage did not participate in the survey and qualitative interviews of the research.

There was no interaction or communication between the observer and the participants during the procedures that required stethoscope use. Participants were not informed about the specific procedure to be observed. Blinding was provided even if participants knew they were being observed. Only after the observation form had been completed were the participants asked to fill in the 4-item “knowledge and attitude form”. This sequence was maintained to ensure that the this did not affect their practices. Twelve participants declined to share their observation results, and were thus not included in the study. Filling out the knowledge and attitude form took an average of 10 minutes. Verbal consent to not provide information to the other participants about the study was obtained in order to prevent the participants from influencing one another.

The data were collected during the working hours from 08:00 to 17:00, when the participants were working as a team. If they were on annual leave or away from the hospital, data were collected on the first day they started working.

The authors individually contacted the nurses working in different clinics and gave verbal information about the study. Semi-structured in-depth interviews were conducted based on phenomenological methods. The identities of the participants and the clinics where they worked were kept confidential and the participants were notified about this. The interviews were held where the participants felt comfortable (e.g., nurses’ room, staff room).

In the interview, data were collected from the participants in a single session. The interviews continued until data saturation was reached. The interview with the participants lasted 45–60 min. In the interviews, questions prepared by the researchers were asked and then the participants were asked to explain their answers in detail by asking in-depth questions such as: *“Can you tell me more?”*, *“What did you mean?”* or *“How?”* Audio recordings were made of the interviews, and were transcribed at the end of the interviews. The participants’ tone of voice were recorded by the interviewer during the interviews. After the interview, nurses were given the opportunity to review the audio recordings to verify the transcript of the interview.

### Data analysis

Qualitative interview results were analyzed through reflexive thematic analysis (RTA) as described by Braun and Clarke [26]. In the first step, two researchers read and re-read the text to ensure correctness and exclude typing errors. In addition, the researchers discussed the transcribed content. The first impressions and the perceived similarities and differences were recorded in the second step. In the third step, the data were systematically divided into meaningful codes. In the fourth step, these initial codes were noted and re-viewed; thus, codes became visible. In the fifth step, the coded data were advanced into a thematic map-making where the researchers considered the adjustment of themes and sub-themes. In the sixth and final step, each theme was analytically refined and related to the literature, and evident definitions were made for each theme and sub-themes. The MAXQDA 10.4 program was used for the analysis and coding of the qualitative interviews.

The analysis of the quantitative data obtained from the research was carried out using SPSS (Statistical Package for Social Science) 21.0. In the data analysis, n, %, and means were used for descriptive statistics. Numerical and percentile distribution were used in the analysis of the data. 

## Results

### Quantitative results

80.7% (n=163) of the participants were female, and 75.7% (n=153) were undergraduates with a mean age of 38.15±2.76 years. 86.4% (n=171) stated that stethoscope disinfection should be done by the people using it and 74.2% (n=150) of the participants stated that stethoscope disinfection should be done before and after contact with the patient (Table 1 (Tab. 1)).

In order to determine their knowledge and attitudes towards stethoscope disinfection, a 10-item Likert scale was employed. Each item was scored as strongly agree (5), agree (4), undecided (3), disagree (2) and strongly disagree (1). The highest possible score was 50. the mean total score of nurses’ knowledge levels and attitudes about stethoscope disinfection was 40.68±2.91 (Table 2 (Tab. 2)).

It was observed that 23.7% (n=48) of the nurses disinfected their stethoscopes before contact with the patient, 11.8% (n=24) after contact with the patient, and 6.4% (n=13) both before and after contact with the patient. The nurses used a stethoscope on an average of 7.42 patients without disinfecting it. 52.8% (n=38) of the nurses who disinfected the stethoscope did so with ethanol based disinfectant (Table 3 (Tab. 3)).

### Qualitative results

**Figure 1:**
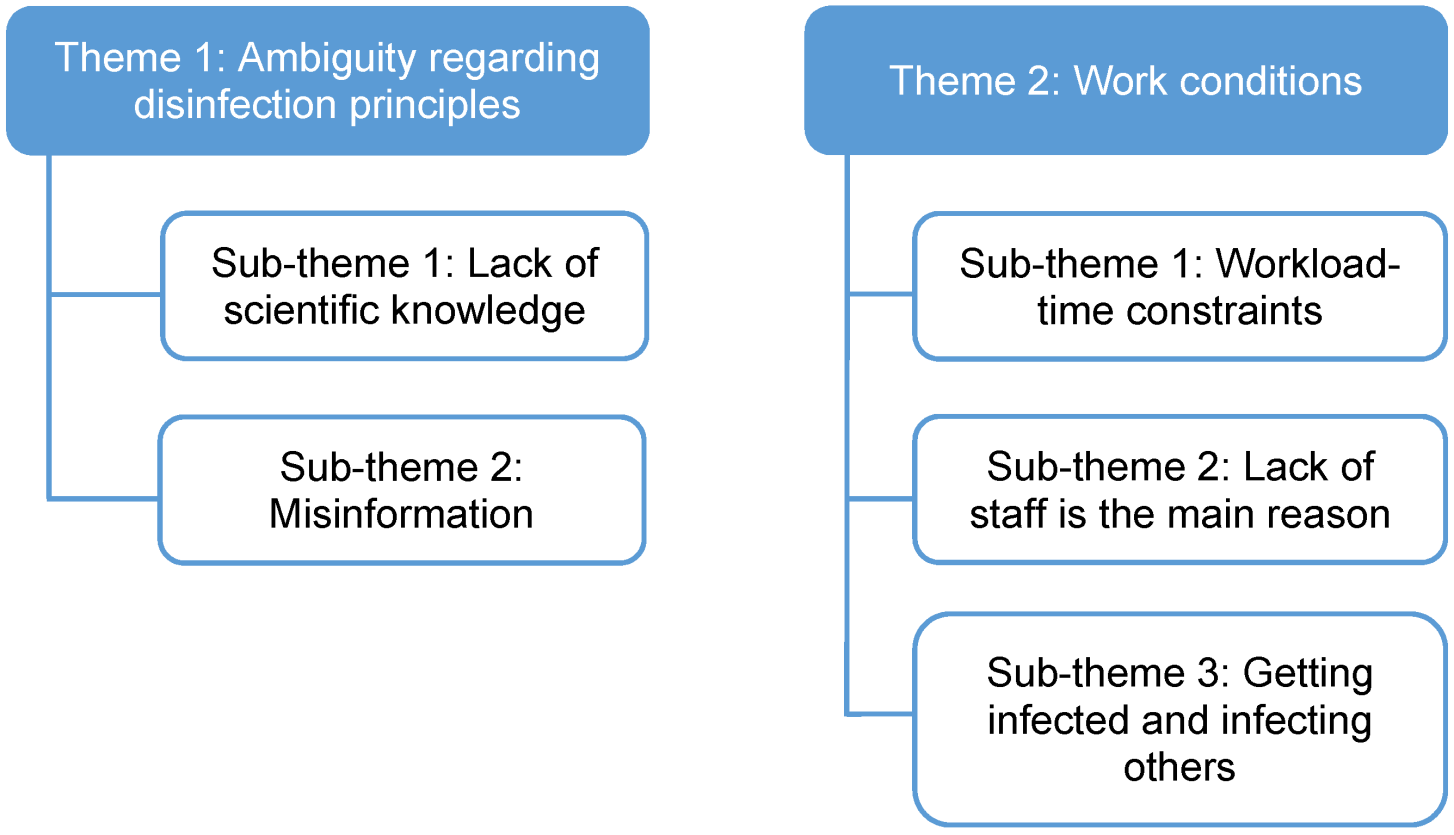
Themes and sub-themes

Themes and sub-themes are presented in Figure 1 (Fig. 1).

### Theme 1: Ambiguity regarding disinfection principles

#### Sub-theme 1: Lack of scientific knowledge

Stethoscopes are included in the category of non-critical medical devices. However, the participants mentioned that they did not know in which category stethoscope disinfection belonged. Moreover, they reported lack of awareness about the existence of a guideline for stethoscope disinfection (see their answers below). 

“I don’t know where to specifically find information on disinfection stethoscopes. I’ve never been trained in this before… I don’t know if there is such a guideline. Especially when I feel that the stethoscope is dirty during the changing of the shift, I disinfected it with any disinfectants that I can easily reach, such as alcohol or hand sanitizer. I had never thought about this before” (N6).

“I have my own stethoscope and I use it all the time. Since no one else uses it, I don’t disinfect it as often. I know it needs to be disinfected but I don’t do it regularly. I know the general disinfection principles, but I don’t know about a stethoscope-specific disinfection. I need to do some research about this” (N10).

#### Sub-theme 2: Misinformation

Especially the nurses who recently graduated reported that they received information regarding disinfection from the nurses they worked with throughout their training and applied this knowledge to their practice (see their answers below).

“I have been working in the clinic for about 2 years. When I was a student and since I started working, I haven’t seen many nurses in my team disinfect their stethoscopes. When I first started to work, I disinfected it every time I came on my shift and used the stethoscope, but now I remember that experienced nurses said that you don’t need to disinfect that much… I think I gave up this habit over time” (N2).

“I disinfect my stethoscopes with alcohol, but I am not sure about the effect on the microorganisms that grow on it, but I think it is disinfected” (N8).

### Theme 2: Work conditions

#### Sub-theme 1: Workload-time constraints

The participants stated that they did not have time for stethoscope disinfection, because they had limited time to deal with patients in inpatient services and polyclinics, and the number of patients per nurse has increased recently (see their answers below).

“I work alone on my shift and I have a lot of work to do. I am racing against time to take patients’ vital signs and administer treatments. That’s why I don’t have time to disinfect the stethoscope while moving between the patient rooms... Unfortunately” (N1).

“I am actively working in the polyclinic. There are many patients, I need to use time effectively to see all of them. I look after patients one after another. In the meantime, sorry but I don’t have time for this.” (N3)

#### Sub-theme 2: Lack of staff is the main reason

The participants considered the high number of patients and the low number of staff as the reason for every negative outcome. They emphasised that more attention could be given to many situations such as disinfection if the number of staff were increased (see their answers below).

“We are very few in number and we cannot keep up with the work. We work as a larger team during the day shift, so I have the opportunity to disinfect my stethoscope during the day, but it is not possible to do it at night. If there are enough nurses to take care of the patients, I would really like to disinfect the devices for both ourselves and our patients, but I can’t keep up with all of them.” (N5)

#### Sub-theme 3: Becoming infected and infecting others

The participants were aware that the stethoscope was a soure of infection and that it was a risk for both healthcare professionals and patients (see their answers below).

“The stethoscope is in contact with many places in different places, going from one patient to another. Of course it’s a big source of infection. Sometimes I use the same stethoscope on too many patients. It is uncomfortable to wear the stethoscope around my neck and continue working, especially after patients with an unknown diagnosis. I’m sure it’s a huge risk. I am aware that I am putting myself and my patients at risk. That’s why I usually disinfect the stethoscope before and after using it” (N11).

“Especially after the pandemic, one of my nightmares… I don’t want to go through the same process again, so I’m afraid of everything that has a contagious effect and I try to take precautions. All nurses were using the same stethoscopes, but we started to limit this practice with the pandemic, now we are trying to bring our own stethoscopes” (N12).

## Discussion

Stethoscopes are among the devices which are potentially contaminated with pathogens and are always in contact with patients, physicians and nurses [27]. Studies showed that 50% to 60% of stethoscopes had bacterial contaminations [2], [28]. For this reason, it is reported that disinfecting stethoscopes before and after contact with the patient will prevent contamination and hospital-associated infections [29].

In the present study, nurses (40.68) scored high on the knowledge and attitude form. Observing stethoscope disinfection frequency of nurses showed that 23.7% of nurses disinfected their stethoscopes before contact with the patient, 11.8% after contact with the patient, and 6.4% of nurses disinfected their stethoscopes before and after contact with patient. Birlie et al. [12] found that about 2.9% of the stethoscope disinfection practices were done before meeting the patient and 1.5% after meeting the patient. In another study, it was stated that 2.8% of the participants disinfected stethoscopes. Moreover, in cases where disinfection was performed, only 4% of the cases complied with the CDC guideline [29]. A study of doctors revealed that just 13.9% disinfect their stethoscopes after each use. The research also discovered that the primary reasons for this low disinfection rate were a lack of disinfectant in the workplace, forgetfulness, and negligence [30]. In another cross-sectional survey of 358 healthcare providers (attending physician, medical students, nurses, and residents), 76% used their stethoscopes frequently at work. Although 93% of them were aware of the possibility of pathogen transmission via stethoscope, only 29% reported stethoscope disinfection after every use [10]. In a different study, stethoscope disinfection was observed in just 2% of cases before patient contact and 16.3% after patient contact. It was also reported that in 90.4% of the cases, the disinfection time was less than 15 seconds [8]. In another study, it was found that 53.2% of healthcare workers never disinfected their stethoscopes, 24.2% had disinfected their stethoscopes more than 8 weeks ago and only 9.6% disinfected had their stethoscopes less than 1 week ago [31]. According to these results, it can be argued that the results of this observational study are similar to the studies in the literature. Although the knowledge scores of nurses regarding stethoscope disinfection were high, the observations made in the clinic pointed to the opposite. Additionally, there are limitations in this part of our study. The nurses using stethoscopes could be observed only at certain hours. The status of stethoscope disinfection was not recorded when the nurses were not observed during the full work day. In stethoscopes that came into contact with the patient, we could not observe findings such as infection or open-wound bleeding. Although this was observed in some cases with some patients, some of the participants were observed to put stethoscopes in their apron pockets or hang them around their necks. This practise may have caused contamination. It may be recommended to work on all surfaces with which stethoscopes come into contact should be examined to determine the source and rate of bacterial colonisation.

Particularly in our study, all of the nurses stated that they thought stethoscope disinfection needed to be done regularly. Likewise, in the study by Peacock et al. [31], most healthcare workers stated that stethoscopes should be disinfected. However, the results of the literature and our results are quite incongruent. It is very likely that there is a gap between nurses’ level of knowledge about stethoscope disinfection and their practices. For this reason, we investigated this issue in depth in the qualitative data section in order to explain the nurses’ lack of practice regarding the disinfection of stethoscopes.

Nurses were asked about the barriers regarding stethoscope disinfection. Some of the participants in the present study stated that they did not receive training on stethoscope disinfection, and/or they did not know guidelines about it existed. In this situation, we recommend the creation of in-depth guidelines for the disinfection of devices such as stethoscopes and informing nurses about these guidelines. The guidelines can be posted in places where nurses can easily see them in the clinics where they work and can be used as a reminder . In the meantime, the importance and frequency of stethoscope disinfection should be explained to nurses and stethoscope disinfection should be demonstrated practically. Misinformation, workload and insufficient number of nurses, which nurses stated as obstacles, should also be critically examined. Because even if nurses are trained about stethoscope disinfection, it is apparent that they cannot perform it if they cannot find a suitable time for disinfection. For this reason, the qualitative findings of the study should be communicated to nurse managers and necessary measures should be taken to solve these problems.

Based on the qualitative interview results, some nurses stated that they did not have information about the disinfectants to be used in stethoscope disinfection and their effectiveness. In a study of doctors, stethoscope disinfection after each use was significantly correlated with the availability of disinfectants in the workplace [30]. In one study [32] involving doctors and nurses in a hospitial, a training workshop was given on disinfection of stethoscopes and how they can cause hospital-associated infection. The results were intended to motivate towards improving the practice of disinfecting stethoscopes. As part of the training, 70% propan-2-ol units for disinfecting the stethoscope were purchased and placed at strategic medical care points within the hospital. It was observed that some healthcare workers had never performed stethoscope asepsis before the implementation of the workshop. In that study, the contamination rate of stethoscopes decreased from 78.5% to 20.2% after the training and interventions [32]. In prior studies, healthcare providers have suggested that failure to perform stethoscope hygiene is a result of a lack of readily available materials, an absence of visual reminders, concern about stethoscope damage, and a lack of time. All factors potentially improved by a touch-free device that dispenses single-use, disposable plastic covers (barries) for stethoscope diaphragms [11]. We have already mentioned the importance of training nurses in stethoscope disinfection. However, this may not be possible in resource-limited settings. Therefore, it is absolutely crucial to ensure the availability of disinfectant solutions as a prerequisie for positively impacting disinfection compliance. Therefore, the hospital’s purchase of items to be used in stethoscope disinfection and placing them in easily accessible places for nurses will encourage them to increase stethoscope disinfection.

In qualitative interviews with nurses, one of the nurses expressed that when she started working, she disinfected her stethoscope when she came on duty and every time she used it, but later she stopped this disinfection habit. In a study conducted with physicians, it was found that the contamination level of stethoscopes increased with increasing clinical experience/medical education. The contamination of medical students’ stethoscopes was less than the contamination of residents’ stethoscopes, which, in turn, was less than that of attending physicians. This study demonstrated a better habit of disinfection among the students than among trained professionals [33]. This phenomenon is reflected by the qualitative results of our study, in which novice nurses were influenced by the experience and advice of nurses with more years of experience. Experienced nurses should be positive role models for novice nurses. For this reason, experienced nurses and head nurse of station should be targeted as the priority group for training on stethoscope disinfection.

In the qualitative interview, a participating nurse stated that her sensitivity to the risk of contamination increased after the pandemic and that she was adamant about stethoscope disinfection in order not to endanger herself, her colleagues and patients. In particular, it is reported to have significant impact on the nosocomial transmission of COVID-19. It was stated contamination of stethoscopes with SARS-CoV 2 and contact with too many patients may have increased the infection risks [13], as SARS-CoV-2 can survive on steel and plastic surfaces for 72 hours or more [34]. The analyses of 22 studies also reveal that Severe Acute Respiratory Syndrome (SARS) coronavirus, Middle East Respiratory Syndrome (MERS) coronavirus, or endemic human coronaviruses (HCoV) persist on inanimate surfaces such as metal, glass or plastic for up to 9 days [35]. Given the potential for coronavirus to survive for long periods of time on different surfaces, a contaminated stethoscope could jeopardize the safety of patients and healthcare professionals. 

In the present study, it was observed that 52.8% of the nurses disinfected the stethoscopes with alcohol. An observational study on physicians’ and nurses’ stethoscope disinfection practices reported that 7.9% of physicians disinfected the stethoscope with cotton ball dipped in ethanol based disinfectant, 4.8% with hand towel and water, and 15.4% of the nurses disinfected the stethoscope with cotton ball dipped in alcohol [9]. In another study, 67.0% of nurses stated that they disinfected stethoscopes with alcohol [10]. Disinfection of stethoscopes with isopropyl alcohol is recommended and may be effective at eliminating many pathogens [14], [15]. However, even though stethoscope disinfection guidelines and recommendations from the CDC are available (i.e., continuous wiping with isopropyl alcohol for at least 60 s), the overall compliance of health care professionals (HCPs) in daily clinical routine is reported to be extremely low [7], [29]. Although the number of participants who disinfected their stethoscopes was small, most of the nurses who disinfected their stethoscopes did so in accordance with the CDC guidelines.

In addition, this study observed the number of patients treated by nurses who had not disinfected their stethoscopes: nurses contacted an average of 7.42 patients before disinfecting their stethoscopes. A recent review of 28 studies highlighted that 85% of stethoscopes are contaminated with bacteria, including pathogens, and that after just one physical examination, stethoscope contamination is similar to or greater than that of parts of the dominant hand of the examining physician. Countless patients are auscultated daily in emergency rooms, internal medicine wards and general practitioners’ offices. Thus, a given instrument comes into contact with the skin of many patients [6], [36], [37]. According to these results, it can be argued that stethoscopes may contaminate patients, which puts patients and healthcare professionals at risk. This result of our study is crucial; the authors suggest that future studies determine the rates of HAIs that may develop in relation to non-disinfected stethoscope contact with patients.

Alternative strategies to enhance stethoscope hygiene have been limited so far [38]. However, due to the pandemic, the search for new stethoscope protection technologies and concepts has recently started to receive increased attention. Microbiological barriers and covers that prevent direct contact of the stethoscope diaphragm with the patient have been developed [13], [39] and are commercially available, but they must change after each patient and are less sustainable than alcohol based disinfectants. It is also important to make sure that these barrier covers do not interfere with the stethoscope’s ability to effectively transmit sound to the examiner [8]. Despite commercial availability, not all healthcare organizations or institutions in the world have access to them. Such healthcare institutions must overcome the obstacles ensuring the disinfection of stethoscopes and prioritize initiatives to ensure effective stethoscope disinfection.

The present study has several strengths. For instance, participating nurses working in different clinics were observed, instead of including nurses affiliated with only a single clinic. A mixed method was used in by providing a questionnaire to assess the knowledge and attitudes of the participants, making observations, and conducting a qualitative interview to identify barriers to stethoscope disinfection. This mixed method was believed to be more powerful and able to obtain more information than the studies on stethoscope disinfection alone. At the same time, our study revealed the incongruity between knowledge and attitudes of the nurses as reflected in the survey questions and their actual practicesIn this study, we did not influence the behavior of the participants, since we did not provide information about the study subject, even if the participants realized that they were being observed. 

This study also had some limitations. Although observation was intended to be performed without the nurses’ awareness, it is possible that some nurses noticed they were being observed and altered their behavior. There is also a possibility of reporting bias, if participants feared social judgments and thus tended to give more socially and professionally acceptable responses despite assurance of anonymity.

## Conclusions

The participating nurses performed very little stethoscope disinfection before and after contact with the patient. Although their knowledge scores regarding stethoscope disinfection were high, for the various reasons mentioned above, their clinical implementation of such knowledge was low. Thus, we recommend the creation of extended guidelines for the disinfection of devices such as stethoscopes, informing nurses about these guidelines. In the interim, the qualitative findings of the study should be communicated to nurse managers, and nurses should receive practical demonstrations on stethoscope disinfection to reinforce its importance.

## Notes

### Authors’ ORCIDs

- Seda Şahan: 0000-0003-4071-2742
- Sevil Güler: 0000-0002-1707-7333
- Emine Korkmaz: 0000-0001-7801-016X

### Competing interests

The authors declare that they have no competing interests.

## Figures and Tables

**Table 1:**
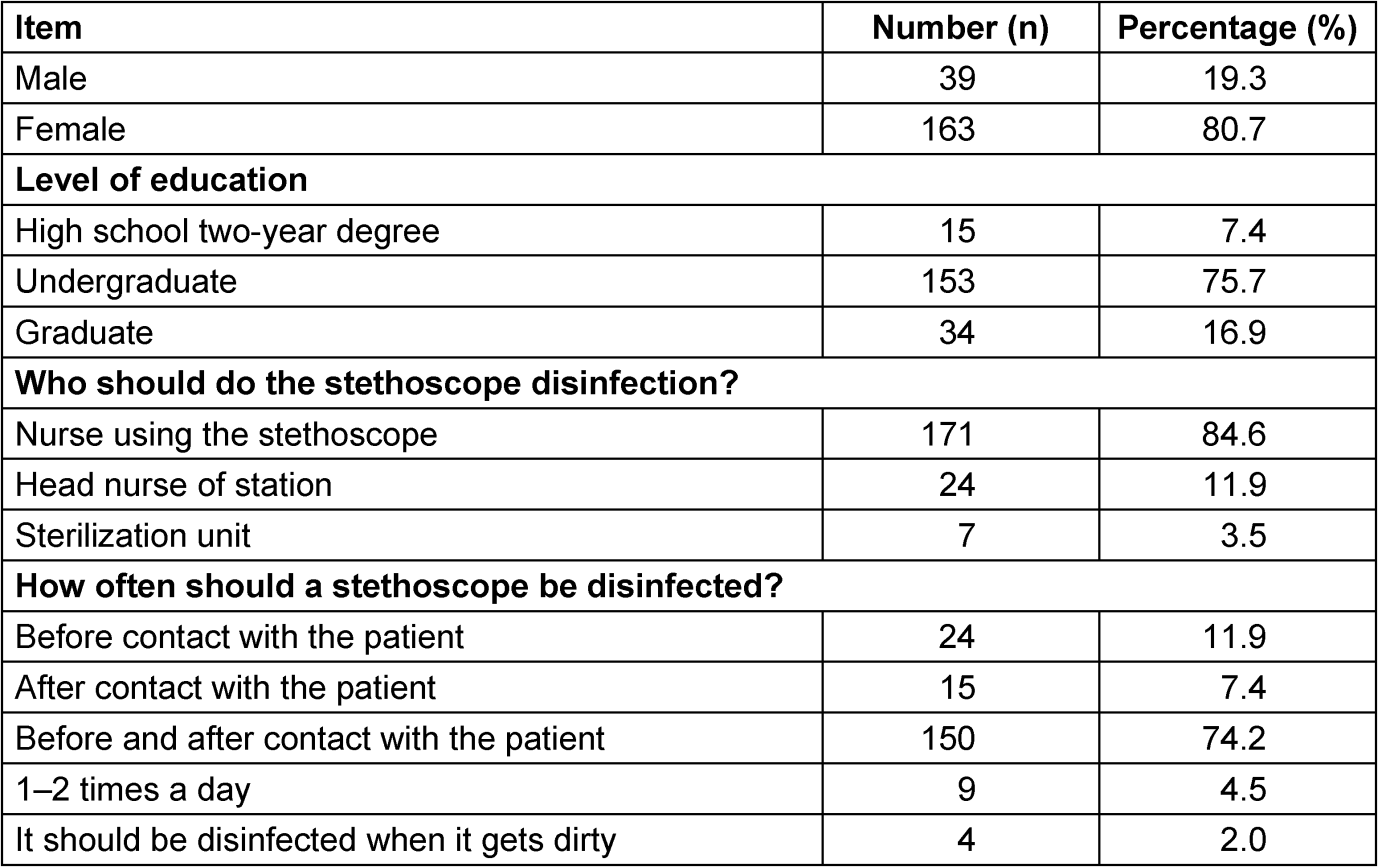
Demographic characteristics of the participating nurses

**Table 2:**
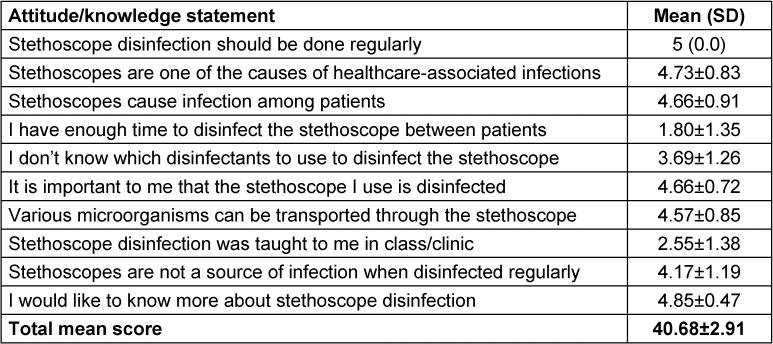
Nurses’ knowledge levels and attitudes about stethoscope disinfection

**Table 3:**
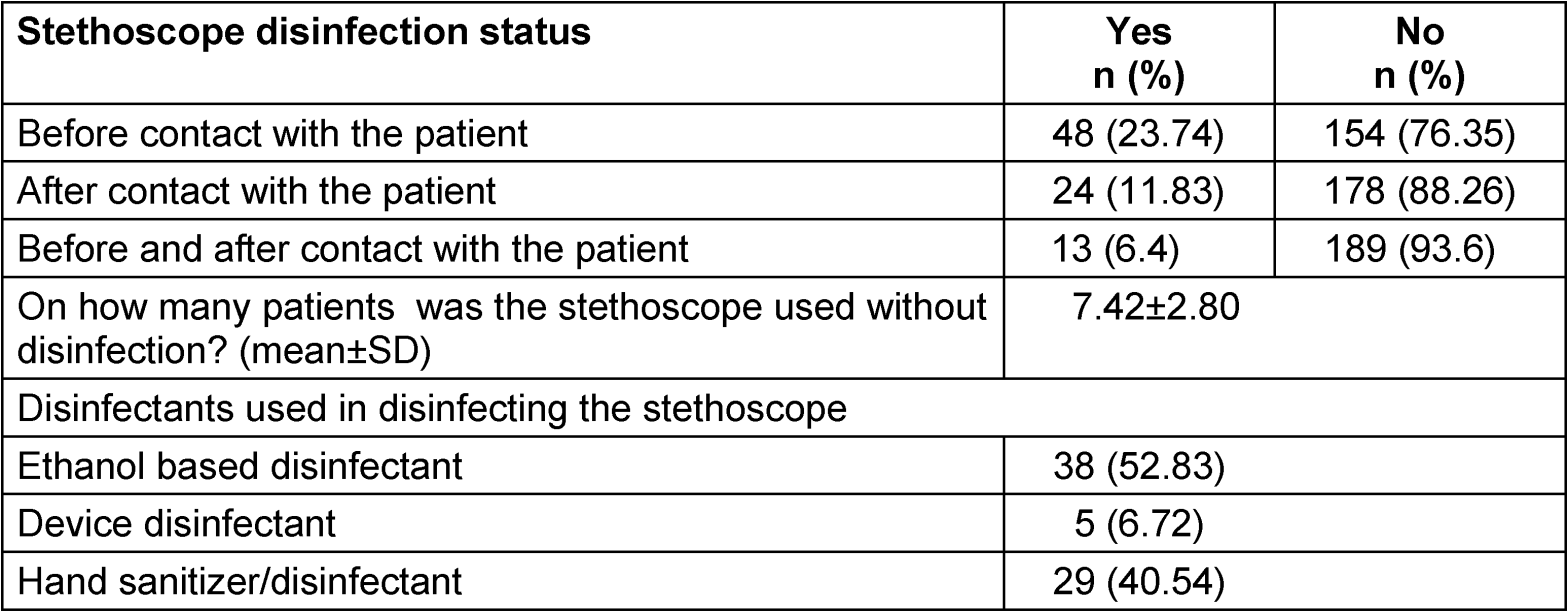
Observation results of nurses’ stethoscope disinfection
